# Invasive pituitary carcinoma associated with antiphospholipid syndrome: a rare case revealed by postpartum cerebral venous thrombosis (case report)

**DOI:** 10.11604/pamj.2026.53.42.50607

**Published:** 2026-01-29

**Authors:** Zakaria Chandide Tlemcani, Driss Said, Hassan Mounir, Jawad Laaguili, Abad Cherif Elasri, Miloudi Gazzaz

**Affiliations:** 1 Department of Neurosurgery, Mohamed V Military Teaching Hospital, Rabat, Morocco

**Keywords:** Pituitary carcinoma, antiphospholipid syndrome, endoscopic transsphenoidal approach, case report

## Abstract

Pituitary carcinoma is an exceptionally rare and aggressive tumor, often diagnosed late due to nonspecific symptoms. Its presentation as postpartum cerebral venous thrombosis is extremely uncommon, and the coexistence of antiphospholipid syndrome further complicates diagnosis and management. We report the case of a 30-year-old woman with a history of recurrent miscarriages who presented 45 days postpartum with severe intracranial hypertension syndrome. Brain magnetic resonance imaging (MRI) with venous MR angiography demonstrated thrombosis of the right lateral sinus extending into the internal jugular vein, along with an invasive intra-sellar mass compressing the optic chiasm and filling the sphenoid sinus. Endoscopic endonasal transsphenoidal surgery allowed partial tumor resection with optic pathway decompression, and histopathology confirmed pituitary carcinoma. The etiologic workup identified positive antiphospholipid antibodies. Management included full-dose anticoagulation and close neurological, endocrinological, and hematological monitoring. This report illustrates a rare and diagnostically challenging coexistence of antiphospholipid syndrome and invasive pituitary carcinoma. It highlights the diagnostic complexity and the need for rapid, multidisciplinary management in atypical presentations.

## Introduction

Pituitary carcinoma is an exceptionally rare and aggressive malignancy, with fewer than 100 cases reported. Its nonspecific presentation often delays diagnosis, and its invasive behavior contributes to significant morbidity [1]. Antiphospholipid syndrome (APS) is an autoimmune prothrombotic disorder responsible for recurrent thrombosis and pregnancy complications [2]. It is a recognized cause of cerebral venous thrombosis, particularly in postpartum women. The coexistence of APS with an invasive pituitary carcinoma is extremely uncommon and poses important diagnostic and therapeutic challenges.

## Patient and observation

**Patient information:** we report the case of a 30-year-old woman with a history of three early miscarriages. She was admitted 45 days postpartum with severe right-sided headaches, blurred vision, and ipsilateral cervical fullness.

**Clinical findings:** ophthalmologic evaluation revealed decreased visual acuity (5/10 in the right eye and 6/10 in the left), right ptosis, impaired abduction of the left eye, and bilateral grade-2 papilledema, consistent with intracranial hypertension and cavernous sinus involvement.

**Diagnostic assessment:** biological assessment showed markedly elevated prolactin levels (1092 ng/mL), normal cortisol levels, preserved thyroid function, and normal growth hormone levels. Both C-reactive protein (78 mg/dL) and D-dimer levels (25 mg/L) were elevated.

**Figure 1:**
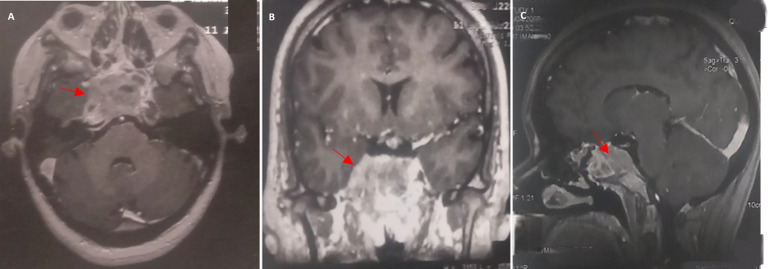
A, B, C) magnetic resonance imaging showing an invasive intra-sellar mass compressing the optic chiasm and completely filling the sphenoid sinus (red arrow)

**Figure 2:**
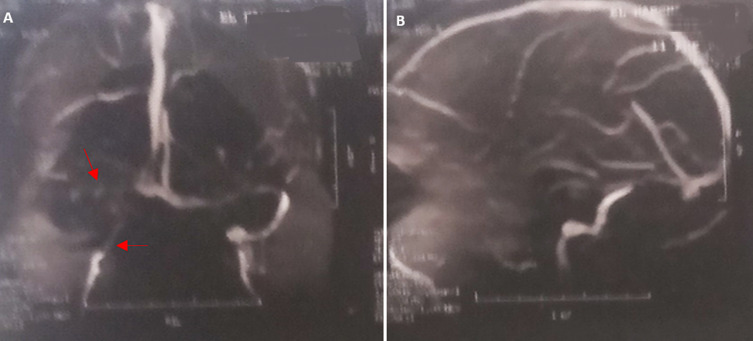
A, B) magnetic resonance venography showing thrombosis of the right lateral sinus extending into the right internal jugular vein (red arrow)

**Figure 3:**
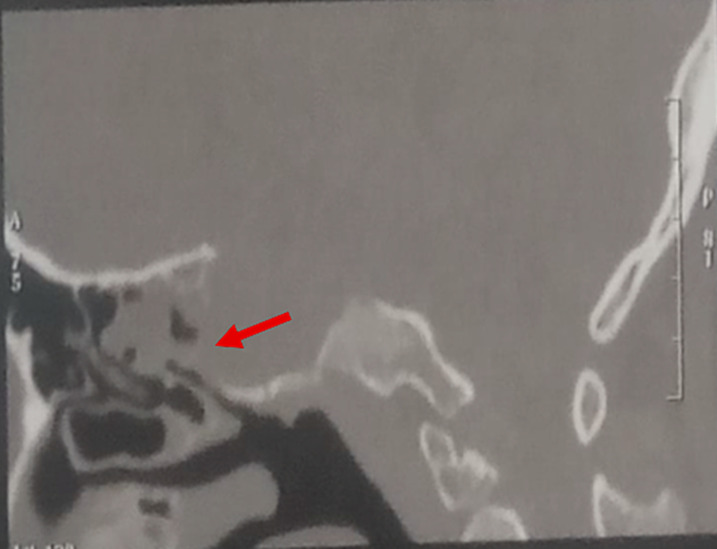
computed tomography scan showing an intra-sellar soft tissue lesion filling the optic chiasm cistern and displacing the optic chiasm superiorly, with involvement of the sphenoid sinus (highlighted by the red arrow)

Brain magnetic resonance imaging (MRI) combined with venous MR angiography demonstrated thrombosis of the right lateral sinus extending into the ipsilateral internal jugular vein. Imaging also revealed an invasive intra-sellar mass compressing the optic chiasm, filling the sphenoid sinus, and eroding the sellar floor, highly suggestive of an aggressive pituitary neoplasm (Figure 1, Figure 2, Figure 3).

**Diagnostic:** therapeutic anticoagulation was initiated, and the etiological evaluation revealed positive anticardiolipin and anti-β2-glycoprotein I antibodies, confirming the diagnosis of antiphospholipid syndrome.

**Therapeutic interventions:** given the visual compromise, an endoscopic endonasal transsphenoidal approach was performed, allowing partial decompressive resection. Histopathological examination confirmed a prolactin-producing pituitary carcinoma, with diffuse prolactin expression on immunohistochemistry, a high Ki-67 index, and p53 overexpression. Cabergoline and temozolomide (TMZ) therapy was initiated, and the patient was managed under close multidisciplinary supervision involving endocrinology, oncology, and intensive care.

**Follow-up and outcome of interventions:** unfortunately, despite adequate high-dose anticoagulation, the postoperative course was complicated by massive pulmonary embolism one week after surgery. She required intubation and intensive care management but died one week later.

**Informed consent:** the study was conducted in accordance with ethical principles. The case report is presented anonymously, and all identifying information has been removed to protect patient confidentiality. Written informed consent for publication could not be obtained because the patient is deceased. According to institutional policy, ethics committee approval and consent were waived for this posthumous case report.

## Discussion

Pituitary carcinoma is an exceptionally rare and highly aggressive malignancy, representing less than 0.2% of all pituitary tumors [3]. In contrast to the typically benign nature of pituitary adenomas, pituitary carcinoma is defined by overtly malignant behavior, including local invasion into adjacent structures such as the cavernous sinus, bony sellar walls, and cranial nerves, as well as the ability to metastasize distantly, most frequently to the central nervous system, bone, lungs, and liver. In the majority of reported cases, pituitary carcinoma develops from a pre-existing adenoma, often a functional subtype, with prolactin-secreting and ACTH-secreting tumors being the most commonly implicated precursors [4].

Antiphospholipid syndrome is an autoimmune disorder characterized by the persistent presence of antiphospholipid antibodies (anticardiolipin, anti-β2-glycoprotein I, lupus anticoagulant), which induce a hypercoagulable state and predispose patients to recurrent venous or arterial thrombosis [5]. APS may be primary or secondary to other autoimmune conditions such as systemic lupus erythematosus [6].

The coexistence of a prolactin-secreting pituitary carcinoma and antiphospholipid syndrome (APS) represents a particularly challenging clinical situation. Prolactin, a cytokine-like hormone, has been implicated in the pathophysiology of several autoimmune disorders, including systemic lupus erythematosus (SLE) and APS [5,6]. In patients with APS, any surgical, tumoral, or inflammatory trigger substantially increases the risk of thrombotic events. This has been illustrated in previously reported cases, including one describing fatal antiphospholipid syndrome following endoscopic transnasal-transsphenoidal surgery for a pituitary tumor [7]. Fatal or catastrophic APS (CAPS) is the most severe form of APS with multiple organ involvement developing over a short period of time, usually associated with microthrombosis. ‘Definite´ and ‘probable´ CAPS have been defined based on the preliminary classification criteria [8,9].

In our patient, who presented with both an invasive pituitary carcinoma and APS, thromboembolic risk was markedly elevated. The carcinoma itself may compress intracranial vascular structures leading to congestion or local thrombosis, particularly in the dural venous sinuses.

Transsphenoidal or transcranial surgery often required in the management of such tumors, carries high risk in APS patients due to the imbalance between thrombotic and hemorrhagic risks. Moreover, adjunctive treatments such as radiotherapy or chemotherapy (notably temozolomide, used in cases of progression or metastasis) [10] may further complicate APS management. Radiotherapy can induce endothelial injury that promotes microthrombosis, while chemotherapy may cause thrombocytopenia and increase bleeding risk, making anticoagulation particularly difficult to balance. Special attention must also be paid to hormonal monitoring, as tumor destruction and therapeutic interventions may lead to hypopituitarism requiring corticosteroid or thyroid hormone replacement. These therapies may, in turn, interact with coagulation pathways and influence treatment tolerance.

Finally, it is important to note that APS can coexist with other autoimmune endocrine diseases, such as Hashimoto thyroiditis or autoimmune insufficiency, further complicating overall management [5]. Close coordination among specialties: endocrinology, neurosurgery, hematology, oncology, and internal medicine is essential to tailor therapeutic decisions according to disease progression and APS-related risks. Although the overall prognosis remains guarded, rigorous multidisciplinary management may allow for temporary stabilization or clinical improvement.

## Conclusion

This case highlights the exceptional presentation of a highly aggressive pituitary carcinoma revealed by postpartum cerebral venous thrombosis. It underscores the importance of considering this rare entity in the differential diagnosis of atypical cerebral venous thrombosis, particularly in the presence of an underlying autoimmune disorder. Prompt, coordinated, and multidisciplinary management is essential to optimize outcomes in such complex and high-risk clinical scenarios.

## References

[1] Heaney AP (2011). Clinical review: Pituitary carcinoma: difficult diagnosis and treatment. J Clin Endocrinol Metab.

[2] Di Prima FA, Valenti O, Hyseni E, Giorgio E, Faraci M, Renda E (2011). Antiphospholipid Syndrome during pregnancy: the state of the art. J Prenat Med.

[3] Shimon I, Melmed S (1998). Management of pituitary tumors. Ann Intern Med.

[4] Du Four S, Van Der Veken J, Duerinck J, Vermeulen E, Andreescu CE, Bruneau M (2022). Pituitary carcinoma - case series and review of the literature. Front Endocrinol (Lausanne).

[5] Rout P, Goyal A, Singhal M (2025). Antiphospholipid Syndrome. StatPearls [Internet].

[6] Fojtíková M, Cerná M, Pavelka K (2010). A review of the effects of prolactin hormone and cytokine on the development and pathogenesis of autoimmune diseases. Vnitr Lek.

[7] Li CZ, Li CC, Hsieh CC, Lin MC, Hueng DY, Liu FC (2017). Fatal antiphospholipid syndrome following endoscopic transnasal-transsphenoidal surgery for a pituitary tumor: A case report. Medicine (Baltimore).

[8] Asherson R, Cervera R, de Groot P, Erkan D, Boffa M, Piette J (2003). Catastrophic antiphospholipid syndrome: international consensus statement on classification criteria and treatment guidelines. Lupus.

[9] Aguiar CL, Erkan D (2013). Catastrophic antiphospholipid syndrome: how to diagnose a rare but highly fatal disease. Ther Adv Musculoskelet Dis.

[10] McCormack A, Dekkers OM, Petersenn S, Popovic V, Trouillas J, Raverot G (2018). Treatment of aggressive pituitary tumours and carcinomas: results of a European Society of Endocrinology (ESE) survey 2016. Eur J Endocrinol.

